# Mycorrhization Mitigates Disease Caused by “*Candidatus* Liberibacter solanacearum” in Tomato

**DOI:** 10.3390/plants8110507

**Published:** 2019-11-15

**Authors:** Eric-Olivier Tiénébo, Kyle Harrison, Kouabenan Abo, Yao Casimir Brou, Leland S. Pierson, Cecilia Tamborindeguy, Elizabeth A. Pierson, Julien G. Levy

**Affiliations:** 1 Department of Horticultural Sciences, Texas A&M University, College Station, TX 77843-2133, USA; eric.tienebo@inphb.ci (E.-O.T.); kharrison@tamu.edu (K.H.); 2 Department of Agriculture and Animal Resources, Institut National Polytechnique Felix Houphouët-Boigny, PoBox 1313 Yamoussoukro, Cote d’Ivoire; a.kouabenan@gmail.com (K.A.); ycasimir.brou@gmail.com (Y.C.B.); 3 Department of Plant Pathology and Microbiology, Texas A&M University, College Station, TX 77843-2133, USA; lspierson@tamu.edu; 4 Departments of Entomology, Texas A&M University, College Station, TX 77843-2133, USA; ctamborindeguy@tamu.edu

**Keywords:** *Rhizophagus irregularis*, *Candidatus* Liberibacter solanacearum, tomato, *Bactericera cockerelli*, mycorrhizae-induced resistance

## Abstract

Disease caused by the bacterial pathogen “*Candidatus* Liberibacter solanacearum” (Lso) represents a serious threat to solanaceous crop production. Insecticide applications to control the psyllid vector, *Bactericera* *cockerelli* Šulc (Hemiptera: Triozidae) has led to the emergence of resistance in psyllids populations. Efforts to select natural resistant cultivars have been marginally successful and have been complicated by the presence of distinct Lso haplotypes (LsoA, LsoB) differing in symptoms severity on potato and tomato. A potentially promising management tool is to boost host resistance to the pathogen and/or the insect vector by promoting mycorrhization. Here we tested the hypothesis that mycorrhizal fungi can mitigate the effect of Lso infection on tomato plants. The presence of mycorrhizal fungi substantially delayed and reduced the incidence of Lso-induced symptoms on tomato as compared to non-mycorrhized plants. However, PCR with specific Lso primers revealed that mycorrhization did not prevent Lso transmission or translocation to newly formed leaves. Mycorrhization significantly reduced oviposition by psyllids harboring LsoA and survival of nymphs from these eggs. However, mycorrhization had no effect on oviposition by psyllids harboring LsoB or the survival of nymphs from parents harboring LsoB. These findings indicate the use of mycorrhizal fungi is a promising strategy for the mitigation of disease caused by both LsoA and LsoB and warrants additional field testing.

## 1. Introduction

Diseases caused by “*Candidatus* Liberibacter solanacearum” (Lso) have had significant economic impacts on potato and tomato production in North and Central America and New Zealand [1,2]. Lso is a rod-shaped, Gram-negative bacterial species belonging to the α-proteobacteria class and the *Rhizobiaceae* family [3]. “*Ca.* Liberibacter” species are generally regarded as important emerging plant pathogens worldwide, and all of them rely on psyllid vectors for transmission [2]. Lso is related closely to “*Ca*. Liberibacter” species responsible for Huanglongbing (HLB), also known as citrus greening disease. These include: “*Ca*. L. asiaticus” (CLas), “*Ca*. L. africanus,” and “*Ca*. L. americanus.”

Six different haplotypes of Lso have been identified to date (LsoA, LsoB, LsoC, LsoD, LsoE, LsoU), which currently are delineated by host, geographic range, and vector species [4,5,6,7,8,9,10]. The best-characterized disease caused by LsoA and LsoB is zebra chip disease of potato (ZC), which results in reduced tuber quality and quantity, and often plant death [11]. LsoA and LsoB cause ZC in the United States and LsoA causes ZC in New Zealand [7,12]. In addition to ZC, LsoA and LsoB cause diseases of other economically important solanaceous hosts, including tomato, pepper, and eggplant [3,13]. Both are vectored by the potato psyllid (also known as the tomato psyllid) *Bactericera cockerelli* (Šulc) [5]. LsoC, LsoD, and LsoE are associated with *Apiacea* crops in Europe, the Middle East, and North Africa [9]. LsoC and LsoD are vectored by the carrot psyllids *Trioza apicalis* and *Bactericera trigonica*, respectively, whereas LsoE has no confirmed vectors [9]. LsoU is associated with *Urticaceae* [9].

Lso is phloem-restricted, causing symptoms typical of phloem-limited pathogens [14]. Aboveground symptoms of Lso infection on potato and tomato include stunted growth (shortened, thickened internodes), leaf symptoms (purpling of lamina or midvein, chlorosis, curling), wilt symptoms (necrosis, plant collapse), and premature death [15,16]. It was observed previously that LsoB causes more significant symptoms than LsoA on tomatoes [17]. Greenhouse-grown tomatoes infected with LsoB typically showed earlier and more severe symptoms, including plant stunting and leaf curling, chlorosis, necrosis, and death of both young and old leaves, and newly produced leaves were often small and discolored [17]. By the eighth week post-infection, greenhouse plants infected with LsoB were typically dead or dying [17]. In contrast, LsoA-infected tomatoes continued to develop new leaves similar in appearance to uninfected plants, but were shorter than uninfected plants and typically remained alive more than 8 weeks post infection [17]. To date, Lso remains non-culturable in vitro [2].

Currently, the most widely used approach to manage Lso-related diseases is early and repeated applications of insecticides [18]. The consequences of these methods are the emergence of resistant psyllid populations [19] and antibiotic-resistant Lso pathovars. Efforts to select natural resistant cultivars have not been successful, but have identified differences in susceptibility in potatoes [20,21]. A potentially promising management approach is the application of beneficial microbes capable of promoting plant growth and natural plant defense mechanisms. Fungi capable of forming arbuscular mycorrhizae (AM) are especially good biocontrol candidates because mycorrhization can both stimulate plant health directly by promoting plant nutrient uptake leading to enhanced productivity [22,23] as well as alter plant susceptibility to certain pathogens, insects (vectors), and the symptoms they cause [24]. For example, improved growth and nutritional status with mycorrhization can compensate for insect feeding damage and facilitate the regrowth of tissues [25]. Improvements in nutritional status also may alter the source-sink nutrient distribution patterns within plants and thereby the suitability of foliar tissues to attackers [26,27,28,29,30,31,32]. Previous studies have shown that mycorrhization generally has a negative effect on herbivory by leaf chewing insects, but may have a positive (most frequent), neutral, or negative effect on phloem feeders such as psyllids [27,29,30,31,33]. A limited number of studies have focused on how mycorrhization affects the progress of diseases caused by phloem-limited pathogens vectored by phloem-feeding insects. Most of these reports focused on diseases caused by phytoplasmas and showed a reduction in disease severity or incidence that may be associated with a decrease in the titer of the phytoplasmas [28,34]. Although analysis of mycorrhizal effects on pathogens may be complicated by the potential impact of mycorrhization on the insect vector and other multi-trophic interactions, AM formation on the rootstocks of grafted tomato and pear also resulted in the reduction of disease symptoms caused by a *Phytoplasma spp.* [28]. It has been suggested that mycorrhization elicits an ISR-type response that primes jasmonic acid (JA) signaling in plants mitigating plant health problems caused by chewing insects and phloem residing pathogens, but potentially making them more susceptible to phloem-feeding insects [28,34]. This effect is referred to as mycorrhizal induced resistance (MIR) [35].

In this study, we test the hypothesis that mycorrhization of tomato by the AM fungus *Rhizophagus irregularis* prior to psyllid infestation is capable of mitigating symptoms caused by LsoA and the more severe LsoB. We hypothesize that mycorrhization alleviates disease symptoms via improvements in plant productivity linked to enhanced nutrient uptake capacity and MIR associated with mycorrhization. We also investigate whether mycorrhization alters insect fertility or fitness that may be linked to a MIR effect. To test these hypotheses, a 2 × 3 factorial experiment was used to compare the health and growth of tomato plants with or without mycorrhization by *R. irregularis,* treated with *B. cockerelli* infected with either LsoA or LsoB, or receiving no insects. Symptom development and plant growth were measured over an 8-week period. Psyllid oviposition and nymph survival also were measured.

## 2. Materials and Methods

### 2.1. Plants

The tomato (*Solanum lycopersicum* L.) cultivar Moneymaker (Thompson & Morgan Inc., Jackson, NJ, USA) was used in this study. Seeds were surface-sterilized in diluted commercial bleach (3%) for 30 min and rinsed twice with sterilized water. Surface-sterilized seeds were pregerminated on water agar for 4–5 days. Pregerminated seeds were transferred to pots (10 × 10 × 10 cm) filled with an autoclaved mix (1:1, v:v) of turface (Turface Athletics MVP, Profile Products LLC, Buffalo Grove, IL) and sand (Brown Play Sand, Quikrete, Atlanta, GA) supplemented 1:100 g:g with slow-release rock phosphate. Plants were grown inside insect-proof cages (Bioquip Inc, Compton, CA) at room temperature with a photoperiod of 16h:8h (light:dark). Plants received 30 mL of sterile distilled water on alternating days and also 30 mL of 0.5 × Hoagland solution twice a week.

### 2.2. AM Fungi and Inoculation

The AM fungus utilized in this study is commercially distributed as Myke^®^ Pro Potato L (Premier Tech, Québec, Canada). One-hundred microliter of Myke^®^ Pro Potato L containing 10,500 viable spores/mL of *R. irregularis* were injected into the soil in the immediate vicinity of the root system of one-week-old plants. The control plants also received a 100 µL aliquot of a filtrate (obtained by 30 µm-sieving of the AMF inoculum in order to provide all nutritional and microbial components of the inoculum other than living AMF). Mycorrhization was quantified on 10 plants chosen randomly per inoculation treatment per experiment at three- and eleven-weeks after inoculation after trypan blue staining [36] as the percentage of the root length colonized [37].

### 2.3. Insects

Currently, there are four different *B. cockerelli* haplotypes in the United States [38], and the Northwestern haplotype was used in this study. Colonies of *B. cockerelli* harboring LsoA or LsoB were previously characterized and maintained as described by Mendoza-Herrera et al. [17]. Diagnostic PCR analyses to detect Lso in *B. cockerelli* were performed for all colonies as described previously [39,40], and confirmed infection status. Three weeks after AM fungus inoculation, three adult male and three adult female psyllids from the confirmed LsoA- or LsoB-harboring colonies were placed on a single leaf inside an “organza-bag” [15] in the middle tier of the shoot system and adult psyllids were removed two days later. Uninfested plants were maintained similarly, but no psyllids were added.

### 2.4. Experimental Design

The experiment consisted of a 2 × 3 factorial completely randomized design with two inoculation treatments: non-mycorrhized plants (NM) and mycorrhized plants (Ri) and three infestation treatments: psyllids harboring LsoA, psyllids harboring LsoB, and uninfested plants (no psyllids). There were 12 replicates of each inoculation treatment, totaling 72 plants (one plant per pot). The entire experiment was replicated twice between May 2015 and June 2016, and all experiments were teminated 8 WAI before the plants outgrew the insect-proof cages.

### 2.5. Assessment of the R. irregularis Effects on Disease Development and Plant Growth

After infestation, the disease symptom development was monitored until 8 WAI. The incidence and severity of foliar disease symptoms were scored 3, 6, 8 WAI based on the 0 to 4 scale, as described in Table 1. For incidence, a rating of 1–4 was scored as symptomatic, and severity estimates were made only on symptomatic plants. Growth measurements were taken at experiment termination on 10 randomly chosen plants per treatment and included root and shoot dry weights, plant height, and number of green leaves.

### 2.6. Detection of Lso on Top-Tier Leaves

In order to detect Lso movement from the site of infection, tissues from newly expanded leaves (mid-vein) were sampled from the top-tier of leaves at 3 and 6 WAI. DNA was extracted as described previously [15]. PCR-based detection was carried out using the Lso TX 16/23 primers to amplify 383 bp of the 16S-23S rDNA intergenic region, as described previously [41,42,43]. This primer set cannot discriminate between LsoA and LsoB. Positive PCR controls consisted of psyllid and tomato genomic DNA previously authenticated as infected by Lso, whereas the negative control was genomic DNA from an Lso-free tomato [15]. The tomato elongation factor-1 (EF1) gene primer set EF1 F/EF1 R was used to control for false negatives in plant samples [40].

### 2.7. Assessment of AM Effect on B. cockerelli Oviposition and Nymph Survival

Two days after infestation, the adult psyllids were removed from the leaves, and eggs were counted. One week later, living nymphs were counted on alternate days. Manipulation of plants for nymph counting (including removal and replacement of organza bags) sometimes resulted in injury or breakage of the leaf. Data from leaves that became injured were excluded. Young adults were removed as they emerged.

### 2.8. Statistical Analyses

For all analyses, data from both experiments were pooled. For percent root colonization, data were log-transformed to ensure normality. A general linear model (ANOVA) and Fisher’s protected LSD were used to analyze transformed root colonization data and disease severity data. For the growth data, missing data were input using the multivariate singular value decomposition (SVD) method. Logistic regression was performed on growth data and showed that all growth variables were significant predictors of treatment effects. Growth data were analyzed using a generalized linear mixed model (GLMM) with Lso treatment, mycorrhizal treatment, and Lso × mycorrhizal treatment as fixed effects, and a Student’s t was used for multiple comparisons. For psyllid life stages (eggs vs nymphs), means were compared at each observation date using the same fixed effects and multiple comparison statistic, and a multivariate analysis of variance (MANOVA) was used for comparisons across dates.

All analyses were performed in JMP^®^ version 14.3 (SAS Institute Inc., Cary, NC, USA, 1989-2019), *P*-value < 0.05.

## 3. Results

### 3.1. Estimation of Root Colonization by AM Fungi Rhizophagus Irregularis

Root colonization by *R. irregularis* was assessed twelve weeks after the inoculum was applied to the plants. Data for root length colonization was slightly, but significantly different among treatments, e.g., 82 ± 0.1 ≥ 75 ± 0.1 ≥ 69 ± 0.1%, for plants infested with LsoB-infected psyllids, no-psyllids, and LsoA-infected psyllids, respectively.

### 3.2. Rhizophagus Irregularis Delays the Development of Symptoms Associated with Lso Infection and Promotes Plant Growth under Disease Stress

Plants that were not infested with psyllids remained healthy during the entire experiment and displayed no disease symptoms. For both mycorrhized and non-mycorrhized plants infested with psyllids no disease symptoms were observed at 3 WAI (Table 2). By 6 WAI, both mycorrhized and non-mycorrhized plants displayed disease symptoms (e.g., distorted growth, especially shortened internodes or stunting), regardless of whether the psyllids harbored LsoA or LsoB. Wilt symptoms were observed first on non-mycorrhized plants (~3 days after 3 WAI). Disease incidence based on symptoms at 6 WAI was 100% for non-mycorrhized plants treated with Lso-infected psyllids compared to 42% for mycorrhized plants treated with Lso-infected psyllids (Table 2). By 8 WAI, disease incidence based on symptoms for mycorrhized plants was 58%, and these results were consistent between experiments. However, there were no differences in the mean disease severity indices for non-mycorrhized versus mycorrhized plants that displayed disease symptoms (Table 2). These data indicate that mycorrhization reduced the incidence of disease, but once mycorrhized plants acquired symptoms associated with Lso infection, the symptoms developed to the same level of severity. Notably across both experiments, four of 14 mycorrhized plants that did not have any symptoms at 6 WAI, developed advanced symptoms by 8 WAI. In general, the symptoms because of LsoB were more severe than those because of LsoA (Table 2).

Lso was detectable via PCR by 3 WAI, and about half of the symptomatic plants were Lso positive. By 6 WAI, 75% of the symptomatic plants tested Lso positive via PCR, but only 50% of symptomless, psyllid infested plants were Lso positive (data not shown).

**Figure 1:**
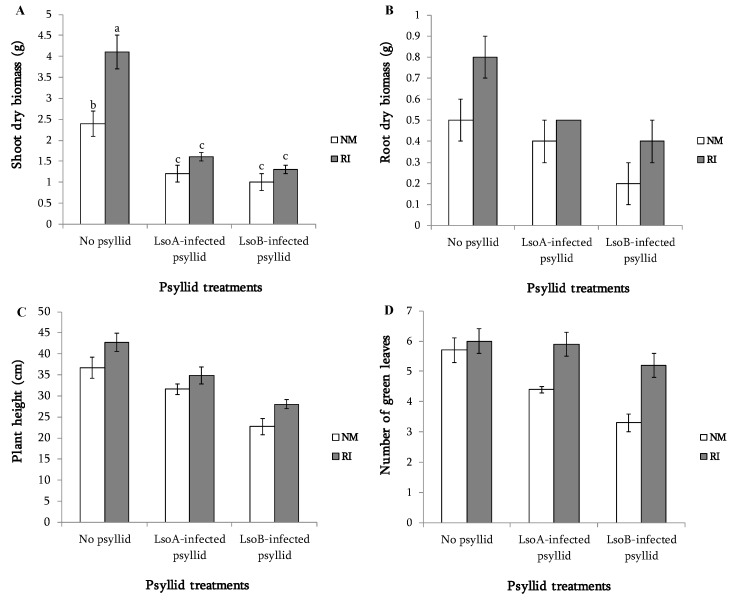
Influence of mycorrhization and Lso infection on tomato shoot dry weight (**A**), root dry weight (**B**), height (**C**), and number of leaves (**D**). NM: non-mycorrhized plants, RI: mycorrhized plants. Growth data are pooled across experiments. For shoot dry weight biomass there was a significant Lso treatment-by-mycorrhization treatment interaction effect, and bars marked with different letters are significantly different (*p* < 0.05) according to multiple comparisons using Student’s t test. For all other variables, there were significant mycorrhization treatment and Lso treatment main effects (*p* < 0.05). Multiple comparisons (*p* < 0.05) were used to test for differences among Lso treatments. Results were as follows: RI > NM, root dry weight: no-psyllid ≥ LsoA ≥ LsoB, plant height: no-psyllid = LsoA > LsoB, leaf number: no-psyllid > LsoA > LsoB.

The analysis indicated a significant Lso treatment-by-mycorrhization treatment interaction effect (*p* < 0.05), but only for shoot dry weight biomass production, indicating that mycorrhization improved shoot biomass production of the uninfected plants, but not the Lso infected plants, which were significantly smaller than the uninfected plants (groups ‘a’ and ‘b’ vs. group ‘c’ in Figure 1A). For all other variables there was a significant main effect for mycorrhization treatment (*p* < 0.05), which resulted in greater root biomass, plant height, and leaf number in mycorrhized plants (NM vs. RI in Figure 1B–D). There also was a significant main effect for Lso treatment for these three variables (*p* < 0.05). Multiple comparisons indicated LsoB-infected plants had less root growth, were shorter, and produced/retained fewer leaves than the uninfected plants, and were shorter and produced fewer leaves than the LsoA-infected plants.

### 3.3. Effect of Mycorrhization on the Development of Nymphs from Parents Harboring Different Lso Haplotypes

Mycorrhization by *R. irregularis* also influenced psyllid oviposition success, but the effect of mycorrhization differed based on the Lso haplotype the psyllids harbored. In the absence of mycorrhization, psyllids harboring LsoA laid significantly more eggs than psyllids harboring LsoB. However, mycorrhization significantly reduced the number of eggs laid by psyllids harboring LsoA, but had no effect on the number of eggs laid by psyllids harboring LsoB (Table 3). All eggs hatched 9 DAI regardless of the treatments. As expected based on egg number, there were more nymphs from LsoA-infected *B. cockerelli* parents on non-mycorrhized plants than mycorrhized plants. However, there was a significant decrease in the number of living LsoA-infected nymphs from 9 DAI to 11 DAI on mycorrhized plants, but no such decline was observed on non-mycorrhized plants. Number of hatched eggs from LsoB-infected parents resulted in statistically similar nymph populations at 9 and 11 DAI, regardless of the mycorrhization treatment. 

The findings indicate that mycorrhization significantly reduced oviposition by psyllids harboring LsoA and survival of nymphs from these eggs. However, mycorrhization had no effect on oviposition by psyllids harboring LsoB or the survival of nymphs from parents harboring LsoB.

## 4. Discussion

Efforts to manage diseases of solanaceous plants caused by Lso have been complicated by the presence of two Lso haplotypes that differ in the intensity of disease symptoms they cause. In this study, we examined whether mycorrhization mitigates disease symptoms in tomato caused by both LsoA and LsoB haplotypes. To avoid bias because of vector genotype, all *B. cockerelli* insects used in this study were from the U.S. Northwestern haplotype. Disease symptoms developed in 100% of non-mycorrhized, psyllid-treated plants. As reported previously [17], disease symptoms in non-mycorrhized plants infected with LsoB were significantly more severe than in plants infected with LsoA. In the previous study, advanced symptom development was associated with higher Lso titer as measured via quantitative PCR and a distinct Lso distribution.

Our study demonstrated that mycorrhization consistently reduced the incidence of disease. Whereas 100% of non-mycorrhized plants developed significant disease, at least 40% of the mycorrhized plants were still without discernable disease symptoms at 8 weeks, and this was consistent in two separate experiments. The benefits of mycorrhization are particularly important for LsoB-infected tomatoes that typically do not survive beyond 8 weeks under greenhouse conditions [17]. We still do not know why some of the mycorrhized plants developed disease symptoms and 40% of the population did not. Our data indicate that mycorrhization did not prevent Lso infection, because Lso was detectable in the non-symptomatic plants. However, as reported previously [15], conventional PCR was not sensitive enough to detect Lso in the newly expanded leaves of all symptomatic plants, and we detected Lso in only ~50% of the Ri-treated non-symptomatic plants. Future studies should investigate whether the reduced disease severity observed in AM inoculated plants is associated with a reduction in Lso titer or a modification of Lso distribution.

Previous reports suggested that mycorrhization may mitigate disease via improvements in plant vigor and MIR-mediated effects on disease progress, psyllid fitness, or both [35]. As expected, mycorrhization promoted shoot biomass production in the absence of Lso infection and root biomass, plant height, and leaf production/retention in the absence or presence of mycorrhization [22,23]. These findings are consistent with the hypothesis that mycorrhization contributed to plant vigor and the mitigation of LsoA- and LsoB-mediated disease symptoms [44]. Enhanced uptake of phosphorous is one of the major benefits provided by mycorrhizae to plants [45], and indeed injection of potassium phosphate (in combination with other compounds) into CLas-infected citrus trees suppressed HLB disease symptoms and reduced CLas titer [46].

A growing body of evidence suggests that mycorrhization elicits a MIR response that primes JA-dependent defense responses in plants [35]. Consistent with this hypothesis, mycorrhization has been shown to mitigate plant health problems caused by necrotrophs and chewing insects, whereas mycorrhized plants may be more susceptible to biotrophs and potentially phloem-feeding insects targeted by salicylic acid (SA) -regulated defenses [28,34]. This pattern correlates with an activation of JA-dependent defenses and repression of SA-dependent ones [35]. JA-dependent defense responses also may reduce insect growth, development, and survival on plants [47]. However, insect behaviors and development on plants are likely to be complicated by the effect of endosymbionts/plant pathogens on insect physiology as well as on JA and SA-regulated plant defenses once transmitted to the plant. Although not investigated in this no-choice experiment, previous studies demonstrated a positive correlation between plant mycorrhization and phloem-feeding performance [27,28,30,31,32,35,48]. We investigated whether mycorrhization would influence other psyllid behaviors. Our study documented MIR-mediated changes in psyllid oviposition behavior, but this effect was strongly dependent on the Lso haplotype harbored by the parent. On non-mycorrhized plants, the number of eggs laid by females harboring LsoA was significantly greater than that observed for females harboring LsoB. On mycorrhized plants, the number of eggs laid by females harboring LsoA was no different from the low number of eggs laid by females harboring LsoB on either mycorrhized or non-mycorrhized plants. Mycorrhization also reduced the survival of nymphs hatching from the eggs of LsoA-infected parents. Nymph numbers from the eggs of LsoB-infected parents were generally low and not significantly different on non-mycorrhized and mycorrhized plants. We do not know the mechanism underlying the effects of MIR on the ovipositioning behavior of parents or the survival of nymphs harboring LsoA. We hypothesize that feeding nymphs might be directly affected by MIR induced production of volatile organics compounds, phytoalexins, or other defense-related molecules produced by plants in response to insect herbivory [49]. We speculate that different Lso haplotypes induce distinct responses in plants and insects, which influence the observed differences in psyllid behavior between LsoA and LsoB parents.

These results constitute the first report that mycorrhization mitigates disease on tomato Lso-caused by both LsoA and LsoB. These results reinforce the idea that mycorrhization confers resistance to phloem-limited pathogens [28,34]. However, Lso-mediated disease involves complex interactions between pathogen, vector, and host, and it is still unclear to us how mycorrhization is affecting these interactions. Future experiments under greenhouse conditions and ultimately in the field will be required to determine whether mycorrhization can protect plants sufficiently to improve marketable yield and to dissect whether there are differences in the outcome of yield trials because of different Lso haplotypes.

## Figures and Tables

**Table 1:** Rating scale of disease severity on tomato.

Score	Symptom Type
0	No symptoms
1	Slight curling and/or purpling of leaves
2	Mild stunting of the plant, wilting and leaf midvein purpling
3	Accentuated stunting, yellowing, interveinal chlorosis. Presence of vein greening mottled or chlorotic leaves
4	Extreme stunting and extreme scorching, wilt, yellowing or interveinal chlorosis. Mottled or chlorotic leaves. Plant collapse and death of the plant.

**Table 2:** Disease incidence and severity assessment.

Disease Quantification	Lso and Inoculation Treatment		3 WAI	6 WAI	8 WAI
**Disease Incidence**	LsoA	Non-mycorrhized plants	0%	100%	100%
		Mycorrhized plants	0%	42%	58%
	LsoB	Non-mycorrhized plants	0%	100%	100%
		Mycorrhized plants	0%	42%	58%
**Disease Severity Indices**	LsoA	Non-mycorrhized plants	0.0	3.0 ± 0.1 b	3.5 ± 0.1 b
		Mycorrhized plants	0.0	3.0 ± 0.1 b	3.7 ± 0.1 b
	LsoB	Non-mycorrhized plants	0.0	3.5 ± 0.1 a	4.0 ± 0.0 a
		Mycorrhized plants	0.0	3.6 ± 0.0 a	4.0 ± 0.0 a

Incidence is reported as the percentage of plants out of 24 replicate plants per treatment that displayed disease symptoms, e.g., disease severity score is ≥ 1. Disease severity was assessed only on plants displaying symptoms and is expressed as the mean ± standard error. Disease incidence was identical across both experiments. Disease severity data are pooled across experiments. For each date, means marked with different letters are significantly different (*p* < 0.05) according to LSD test.

**Table 3:** Psyllid life history assessment.

Treatments		Eggs	Nymphs 9 DAI	Nymphs 11 DAI
LsoA	Non-mycorrhized plants	50.6 ± 3.4 a	37.7 ± 4.3 a	30.5 ± 3.6 a
	Mycorrhized plants	25.1 ± 1.9 b	15.0 ± 2.0 b *	8.2 ± 1.4 b *
LsoB	Non-mycorrhized plants	19.3 ± 2.4 c	6.3 ± 0.9 c	4.9 ± 0.8 b
	Mycorrhized plants	14.9 ± 2.0 c	7.3 ± 1.2 c	6.2 ± 1.1 b

Influence of mycorrhization on oviposition and nymph survival by *B. cockerelli* harboring LsoA or LsoB. Data are pooled across experiments. Number of eggs and the number of nymphs at 9 and 11 days after infestation per leaf. Data are reported as means and standard errors, and for each column means marked with different letters are significantly different (*p* < 0.05) according to Students t test. * indicate a significant change in nymph numbers from 9 to 11 DAI as determined by MANOVA.
